# Nested Pattern Detection and Unidimensional Process Characterization

**DOI:** 10.3390/e26090754

**Published:** 2024-09-03

**Authors:** Gerardo L. Febres

**Affiliations:** Departamento de Procesos y Sistemas, Universidad Simón Bolívar, Sartenejas, Baruta 1080, Miranda, Venezuela; gerardofebres@usb.ve

**Keywords:** patterns, nested patterns, information, entropy, ordered structure, redundancy

## Abstract

This document introduces methods for describing long texts as groups of repeating symbols or patterns. The process converts a series of real-number values into texts. Developed tailored algorithms for identifying repeated sequences in the text are applied to decompose the text into nested tree-like structures of repeating symbols and is called the Nested Repeated Sequence Decomposition Model (NRSDM). The NRSDM is especially valuable for extracting repetitive behaviors in oscillatory but non-periodic and chaotic processes where the classical Fourier transform has limited application. The NRSDM along with the two graphical representations proposed here form a promising tool for characterizing long texts configured to represent the behavior of unidimensional processes.

## 1. Introduction

Patterns are the essence of information. Sometimes established, sometimes waiting to be discovered, patterns comprise a world of interpretations and assigned meanings. Patterns are how nature organizes itself. However, patterns are also the means to interpret and advance our understanding of nature. Patterns are the basis of our knowledge. Number systems assign a sense of quantity to the patterns formed by elementary symbols. Languages consist of rules governing the formation of patterns to create messages, descriptions, and models. Despite the mechanism of associating patterns to produce a communication channel, an abstract perspective is often the approach to study patterns. The semantical meaning, if there is any, is not as important as the quantity of information a pattern can bring with it and how this information quantity varies as the same data set obeys different observation criteria, yielding different interpretations. In 2021, Duin [1] presented an extended treatment of the types of patterns and their crucial role in human knowledge development.

From a formal math point of view, the partitioning of process descriptions into continuous groups was introduced by J. Fieldman [2]. In his work, Feldman studied ergodic theory and proposed methods to assess entropy ergodic transformations. Ergodic theory has evolved as the field from which to study the statistical properties of dynamic systems. Valuable information about ergodic theory’s history and development is found in [3,4].

When the text decomposition is approached as the superposition of several periodic time functions or signals, typical models use sines, cosines, and wavelets as the primal components of the sum. Fourier and wavelet analyses led to the algorithmic cornerstone of the periodic and stationary signal processing tool: the fast Fourier transform (FFT). Nevertheless, nonstationary phenomena escape from the Fourier transform’s capacity to represent them in the domain of frequency. In the comprehensive introduction of their paper, Huang et al. [5] mention the disadvantages of other strategies to decompose a nonstationary series of values. In the same paper, they introduced Empirical Mode Decomposition (EMD), an alternative method to study nonstationary systems that has gained significant popularity in addressing engineering, mathematics, and science problems, as noted by Mahenswasri and Kumar [6]. In the search for a faster method, Neill [7] published a method based on a data sample indicating that potentially relevant patterns are detected. 

Besides the non-periodicity, another aspect that adds difficulty to the series synthesizing process is nested pattern detection. Despite its promises, nested pattern detection poses formidable challenges. The inherent complexity of nested structures demands innovative approaches and specifically developed computerized tools that can disentangle overlapping patterns and discern hierarchical relationships. Detection methods inspect data sets to detect patterns. Traditional methods locate repeated symbolic sequences within a long text. Typically, once the localities of a repeated sequence are determined, the space occupied by these sequences is isolated, and the search for more repeated sequences continues over the remaining text. Thus, the procedure does not further search for repeated sequences within the repeated sequences found; it does not further search for nested repeated sequences. We refer to this type of procedure as the Zero-Depth text interpretation or direct decomposition. In contrast, if the inspection continues looking for repeated sequences within the already found longer sequences, we call the procedure a Nested Pattern Decomposition Model.

This paper explores nested pattern detection comprehensively, leaning on the capacity of a software platform named *MoNet* [8]. *MoNet* comprehends tailored coding pseudo-languages designed to operate with complex data structures as multidimensional orthogonal arrays and data trees. *MoNet* handles these structures as objects allocated in a single memory address, managing them as variables with identity; simultaneously, they can be partitioned according to desired segmenting criteria. *MoNet*’s possibilities for defining math operations using complex structures—which may be considered tensors—as function arguments permit this system to administer multiple complex system experiments within the same environment.

The rest of this document presents two pseudo algorithms for producing the nested repeated sequence decomposition of texts. This decomposition model was applied to three groups of chaotic systems: the May equation, the Lorentz equation, and four stock market indexes. The decompositions obtained allow for estimating the nested complexity of these processes. Finally, we draw some process characteristics from interpreting two graphic representations of the decomposition.

## 2. Interpretation Models for Unidimensional Descriptions: The Sequence Repetition Model

Consider a text (T) formed by a sequence of L elementary characters ($c_{p}$), each referred to according to their position (p) in T. Then, the text (T) can be expressed as the following concatenation of L characters:

(1) $$c_{0}\;\&\;c_{1}\;\&\;\ldots \;\&\;c_{p}\;\&\;c_{p+1}\;\&\;\ldots \;\&\;c_{L-1}\;=\;T.$$

We define the decomposition process as the criteria for forming groups of characters that reduce the T’s complexity. The process inspects the text (T) for sequences of characters that appear $r_{q}$ or more times. The term “symbol” refers to these sequences of characters. However, sometimes we use the term “sequence” to recall the symbols as strings of consecutive characters. Repeated sequences must appear at least $r_{q}$ times. The selection of parameter $r_{q}$ allows the method to be precisely adjusted to the nature and conditions of the text processed. In any case, $r_{q}$ must be equal to or greater than two. Otherwise, a sequence appearing only once is not considered a pattern, and thus, it is regarded as noise. After submitting a text (T) to any process capable of retrieving the repeated char sequences or symbols, we may represent the resulting set (W) of symbols, with their corresponding frequencies of occurrence ($g_{i}$), as follows:

(2) $$W=\left\{{w_{0}:g_{0},\ldots ,w}_{i}:g_{i},\ldots ,w_{u}:g_{u}\right\}.$$

Notice that the series of symbols and frequencies start at index i=0. This means that the number of repeated symbols is u+1. Equivalently, the symbolic diversity of T is u+1.

When interpreting text (T) at its maximum scale, sub-contexts are not considered; thus, there must not be overlapping positions occupied by any two symbols. Consequently, symbols ($w_{i}$) must not share any char position. Interpretations of text (T) may differ when diverse criteria are used to select, within the text, the sets of contiguous characters to form a set of symbols (W). Based on the observer’s choice—or the choice implied by the observer’s criteria—a symbol ($w_{i}$) starting at the text position ($p_{i}$) and made of $l_{i}$ characters is as follows:

(3) $${w_{i}=c}_{i,0}\;\&\;c_{i,1}\;\&\;\ldots \;\&\;c_{i,p_{i}}\;\&\;\ldots \;\&\;c_{i,l_{i}-1}.$$

Additionally, all chars contained in the T are not necessarily part of a symbol ($w_{i}$) in the set (W). The chars not included in any $w_{i}$ are noisy in terms of interpretation. This consideration is consistent with the typical existence of non-synthetized fragments in any system’s description. However, the standard objective of any description is to minimize the noise fraction.

For long texts and time series, the symbol diversity may be significant. In these cases, reducing the complexity of the system’s representation is convenient to make it feasible and controllable for a quantitative analysis. To cope with this, we propose working with the set of symbols grouped by their length. Therefore, we define $Y_{i}$ as the set of all repeated sequences ($w_{i}$) sharing the length $l_{i}$. Correspondingly, let $f_{i}$ be the number of appearances of each symbol category ($Y_{i}$). Thus, the tendency of a process to repeat sequences of values according to their lengths can be characterized by the spectrogram ($\Psi_{f}$), which we define as follows:

(4) $$\Psi_{f}\equiv \left\{{l_{0}:f_{0},\ldots ,l}_{i}:f_{i},\ldots ,l_{q}:f_{q}\right\},$$

where $l_{q}$ and $f_{q}$ are the length and the frequency of the longest repeating sequence within the text (T).

The frequencies ($f_{i}$) are linearly related to the fraction of text (T) occupied by symbols of each length category. Then, by convenience, we alternatively express the characteristic spectrogram ($\Psi_{f}$) in terms of the probability of encountering a $l_{i}$-long sequence within the text (T):

(5) $$\Psi_{p}\equiv \left\{{l_{0}:{Prob}_{0},\ldots ,l}_{i}:{Prob}_{i},\ldots ,l_{q}:{Prob}_{q}\right\}.$$

We refer to the spectrograms $\Psi_{f}$ and $\Psi_{p}$ as the frequency-based and probability-based interpretations. We use the term representation to refer to the selections of char sequences to form sets of symbols (W and Y) obtained after some interpretations.

### 2.1. At-Scale Repeated Sequence Model (ASRSM)

This section aims to build a probability—to be defined by Equations (6) and (7)—model of the repetitions of sequences of similar values in a series and the tendency of the process to repeat same-length cycles. When the original data are a series of scalar numbers, a discretization procedure converts the values into a text formed with an alphabet of as many letters as the resolution of the discretizing process requests (see Appendix A.1 for details).

Developing a procedure to obtain the ASRSM of a long text is an interesting perspective given our expectation of dealing with complex processes that are usually oscillatory but non-periodic. The Discrete Fourier Transform (DFT) algorithm is the classical tool to study oscillatory processes. However, the DFT is not well suited for low-frequency and non-periodic processes [9], and its complexity [10] may represent a barrier with series in the order of thousands of values.

**Figure 1:**
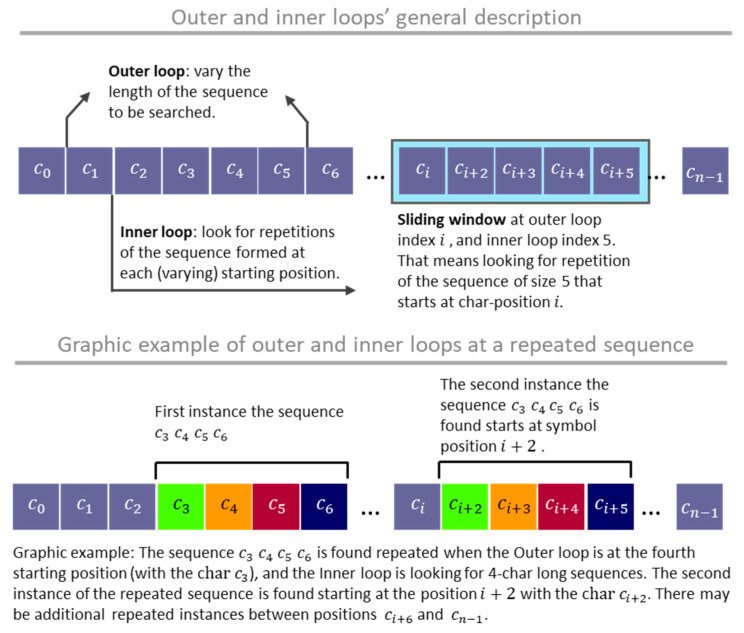
Repeated sequence searching strategy with two nested loops.

The ASRSM identifies repeating sequences within the text (T). To capture the symbol (represented by the repeated sequence) representing relevant information for describing the T, longer repeating sequences are prioritized over shorter sequences. Thus, when looking for the sequences synthesizing a larger process fraction, the ASRSM procedure looks for the longer patterns first. The remaining text is subject to repeated inspections until all the repeated sequences are localized. Figure 1 illustrates the repeated sequence searching strategy with two nested loops. 

**Figure 2:**
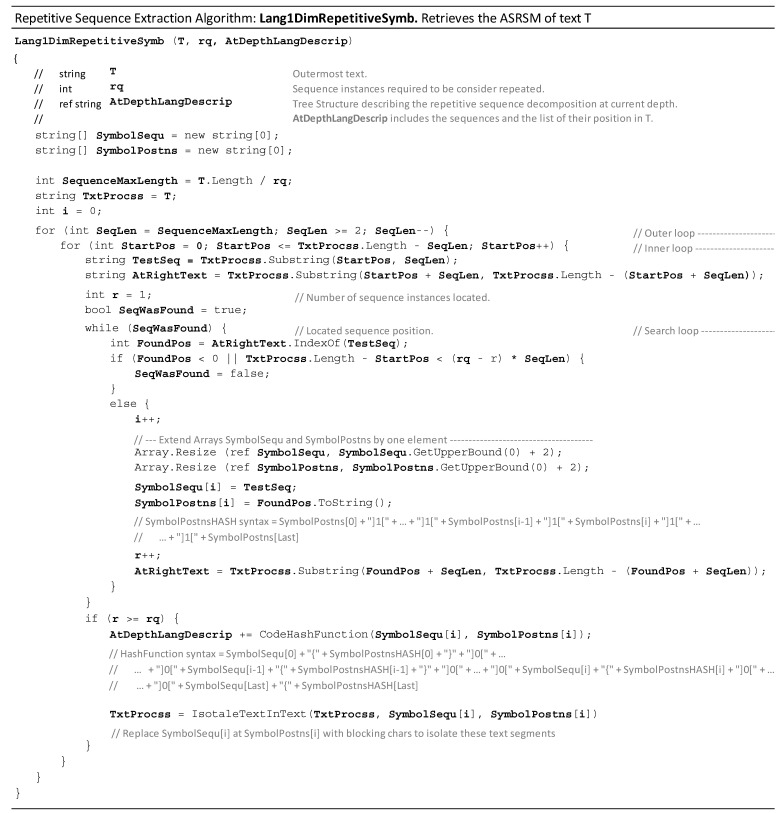
Pseudocode of algorithm **Lang1DimRepetitiveSymb()**: retrieves the At-Scale Repeated Sequence Model (ASRSM).

Figure 2 presents a pseudocode (using the syntax of the C# programming language). The routine **Lang1DimRepetitiveSymb()** retrieves the ASRSM of the text (T). The values of $l_{i}$ and $f_{i}$ corresponding to the most extended repeating sequences detected if T is observed at is largest scale are available from this routine.

Three loops dominate the algorithmic complexity (${AC}_{R}$) of **Lang1DimRepetitiveSymb()**: the Outer Loop, the Inner Loop, and the Search Loop. The number of cycles of the Outer Loop is T.Length/rq. For the Inner Loop, the approximated average number of cycles is .Length − $\frac{T.Length}{2\;*\;rq}$. For the Search Loop, the approximated average number of cycles is $T.Length\;-\left(rq-r\right)*\;SeqLen\approx T.Length\;-(rq-\frac{rq}{2})*\frac{T.Length}{2*\;rq}$. Thus, the Search Loop growth order is O(T.Length). Finally, computing the three loops’ growth order indicates that ${AC}_{R}$ is of the $O({T.Length}^{3})$. The algorithm uses hash procedures to code data structures and pass them as arguments. The complexity of these hash codes grows linearly with the order (O(T.Length)). Therefore, these hash codes do not alter the overall complexity (${AC}_{R}=O({T.Length}^{3})$).

Some properties of the ASRSM are the visible graphing probability vs. the sequence length. We start with a $L_{0}$-character-long text ($T_{0}=T$), formed by s different chars distributed along $T_{0}$. By locating the $l_{0.mx}$-long most extended non-overlapping sequence appearing $r\geq r_{q}$ times in $T_{0}$, we empirically compute the probability of $T_{0}$ containing an $l_{0.mx}$-long sequence that appears $r_{q}$ times. At this point, we introduce the syntax ($Q_{r_{i},l}$) to refer to the likelihood of an l-long sequence appearing exactly $r_{q}$ times in an argument text. After scanning the text and identifying the length of the most extended sequence that appears $r_{q}$ times, we can write the probability of finding the sequence as the fraction it occupies in $T_{0}$. Thus,

(6) $$Q_{r_{q},\;l_{0.mx}}=Q\left(r_{q}times,\;l_{mx}\right)=\frac{r_{q}{\;\cdot \;l}_{0.mx}\;}{L_{0}}.$$

Every time **Lang1DimRepetitiveSymb()** ends the **SearchLoop** (see pseudocode in Figure 2), the locations of the found repeated sequence are locked from the text, leaving a shorter text to be processed. Following the computation of the probability ($Q_{r_{q},l_{0.mx}}$), the search for repeated sequences continues with the remaining text of length $L_{i}$. Identifying the length ($l_{i.mx}$) of the most extended repeated sequences at each search step (i) leads to determining the probability referred to as the length of the remaining text corresponding to the step i. Thus,

(7) $$Q_{r_{i},\;l}=\frac{l}{{\;l}_{i.mx}}\cdot Q_{r_{i},\;l_{i.mx}}.$$

**Figure 3:**
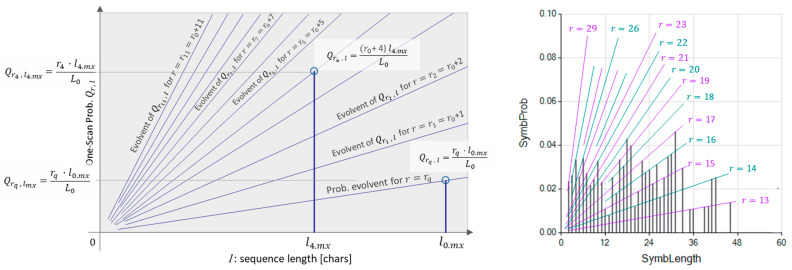
At-Scale Repeated Sequence Model (ASRSM) probability graphs. **Left**: ASRSM probability envelope functions. **Right**: An explicit graphic example of an ASRSM probability for $r_{q}=13$.

The algorithm **Lang1DimRepetitiveSymb()** performs the **SearchLoop** identifying the most extended repeated sequences for $T_{0}$, $T_{1}$, $T_{2}$, and by successively using Equation (7), obtains the intermediate probability ($Q_{r_{i},\;l}$). The final remaining text ($T_{rem}$), not represented by any repeated sequence, is an expression of the noise contained in the original text (T). The values of the probabilities ($Q_{r_{i},\;l}$) exhibit an attractive distribution that is convenient to show here. Figure 3 (left) shows the origin of the ramp-shaped probabilities ($Q_{r_{i},\;l}$). Figure 3 (right) is an actual graph showing the interpretation of the results ($Q_{r_{i},\;l}$).

### 2.2. Nested Repeated Sequence Length Decomposition (NRSLD)

Looking for symbols repeated within another symbol, as in a nested fashion, alters the scope of the observer’s view. Considering “nested contexts” is needed to assess the complexity of a system integrating several scales of observation, thereby revealing the process of the internal structure and allowing for an estimation of the structural complexity that a system (T) represents.

**Figure 4:**
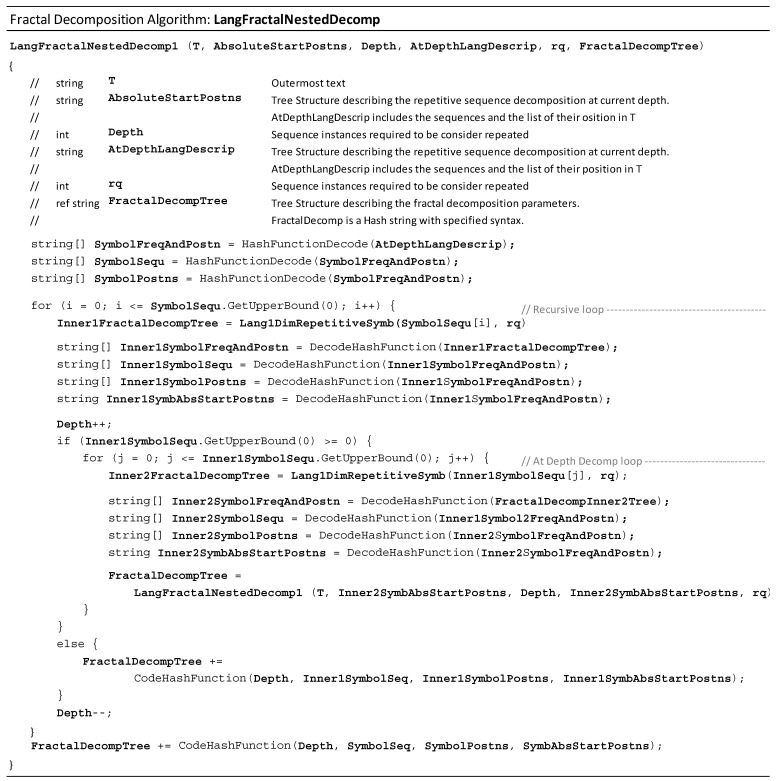
Pseudocode of algorithm **LangFractalNestedDecomp()**: retrieves the Nested Repeated Sequence Decomposition Model (NRSDM).

This section explains a procedure for obtaining these sets of nested sequences, which we name the Nested Repeated Sequence Decomposition Model (NRSDM). The developed routine is named **LangFractalNestedDecomp()**. It recursively iterates over itself and relies on the previously presented procedures, **LangFractalNestedDecomp()** and **Lang1DimRepetitiveSymb()**. Figure 4 presents a pseudocode (using the syntax of the C# programming language). The routine **LangFractalNestedDecomp()** retrieves the NRSDM of the text (T) when observed as a fractal-like structure with sequences compounding at progressively deeper nesting levels.

The algorithmic complexity (${AC}_{F}$) of **LangFractalNestedDecomp()** is estimated considering its two loops: the Recursive Loop and the Depth Decomp Loop. Due to the algorithm’s recursive condition, we can estimate the Depth Decomp Loop cycles as many times as the number of nodes forming the tree **FractalDecompTree**. For a regular tree of D nesting levels (including the root depth and the depth of the leaves), with each node (except the leaves) divided into B branches, the number of nodes is $B^{D-1}$. For instance, assuming a sixth-depth-level regular tree (D=4) where any sequence is depicted by B=9 repeating sequences, the number of steps of **LangFractalNestedDecomp()** is $B^{D-1}=9^{5}=59,049$. Comparing the complexities of routines **Lang1DimRepetitiveSymb()** and **LangFractalNestedDecomp()** leads to the question $O({T.Length}^{3})⩻O(B^{D-1})$. At first glance, the latter seems to be a larger order; however, parameters B and D limit each other. When the length of the repeated sequences found is large, meaning a large B, the depth of the tree, represented with parameter D, tends to decrease. Therefore, in typical situations, we expect $O\left(B^{D-1}\right)<O\left({T.Length}^{3}\right)$, since the spread (B) of the **FractalDecompTree** is considerably smaller than the T.Length, and D is not much larger than 3. Routine **LangFractalNestedDecomp()** starts by decomposing the full text (T) with routine **Lang1DimRepetitiveSymb()**. Even though **Lang1DimRepetitiveSymb()** runs several times afterward, these runs process text lengths that are only a fraction of the initial T.Length. These considerations allow us to state that, in most practical applications, the algorithmic complexity of **LangFractalNestedDecomp()** is ${AC}_{F}=O({T.Length}^{3})$. Finally, the algorithm **LangFractalNestedDecomp()** uses hash functions to code data structures and pass them as arguments. The complexity of these hash codes grows linearly with the Order(T.Length). Therefore, these hash codes do not alter the overall complexity (${AC}_{F}$).

### 2.3. An Example of the ASRSM and the NRSDM of a Tiny Text

**Figure 5:**
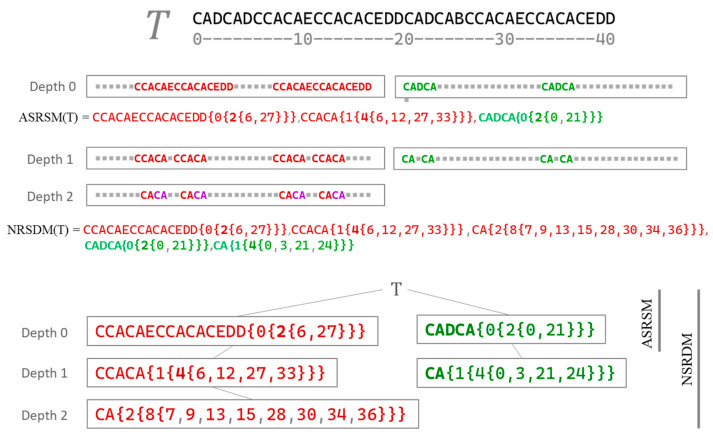
An explicit example of decomposing the text: ***T*** = CADCADCCACAECCACACEDDCADCABCCACAECCACACEDD.

This section presents the ASRSM and the NRSDM describing the four-value discrete-scale text CADCADCCACAECCACACEDDCADCABCCACAECCACACEDD. Obtaining the models uses the procedures **Lang1DimRepetitiveSymb()** and **LangFractalNestedDecomp()**. Figure 5 shows how the text (T) is decomposed by applying procedure **Lang1DimRepetitiveSymb()** at depth zero to obtain the repetitive sequences (ASRSM(T)). In further stages, the method uses **LangFractalNestedDecomp()** to control the recursive application of **Lang1DimRepetitiveSymb()** to obtain the tree-like NRSDM(T). Figure 5 also includes the final ASRSM(T) and NRSDM(T) hash codes.

### 2.4. Graphical Representations of the NRSDM

We present the ASRSM, a procedure conceived to retrieve the longest character sequence that appears at least $r_{q}$ times. Once the text (T) is decomposed, each decomposition element—each repeated sequence found—is subject to the same procedure applied at a deeper nesting level. This recursive nested search repeats through the branches generated whenever the process finds new, more profound repeated sequences. The search for repeated sequences continues until the sequences found do not contain repeated sequences of two or more characters. 

The ASRSM is a valid characterization; however, since every found sequence is isolated from the search space, the ASRSM does not account for the number of times shorter sequences appear contained in longer sequences. Thus, the ASRSM captures the shallow text’s characteristic patterns. To attempt to capture deeper characteristic patterns, we rely on the NRSDM, which not only expresses the tendency of the text-represented process to repeat state sequences but also offers a characterization of the system’s structure by showing how deep a sequence is nested within longer char sequences.

The NRSDM graphically describes a process’s tendency to repeat strings of states—namely, sequences. The potential of this graphic objective, which involves adding attributes like colors and transparencies to the bubbles representing sequences, is promising and fascinating. We may further develop this area in a later paper, sparking intrigue and inspiration, and we look forward to further developing this area in future papers. In this document, we delve into two graphic representations of the NRSDM. The NRSDM produces information about several parameters describing the text (T), which means that the NRSDM is a multidimensional structure that describes the text (T). To appreciate the NRSDM structure in two-dimensional representations, we propose graphing the probability vs. sequence length of the repeated sequences found in the process. Different symbols sharing their lengths are grouped into sets with sequences of the same size. Even though the grouping around symbol lengths reduces the number of points whose probabilities form the graphs, there are still several ways of interpreting these probabilities. We present the following two graphic representations of the NRSDM:

- MSIR: Multiscale Integral Representation. This comprises the sets of symbols sharing their lengths and disregarding the nesting depth where they appear;
- NPR: Nested Pattern Representation. This regards the symbols’ nesting depth within the tree structure.

**Figure 6:**
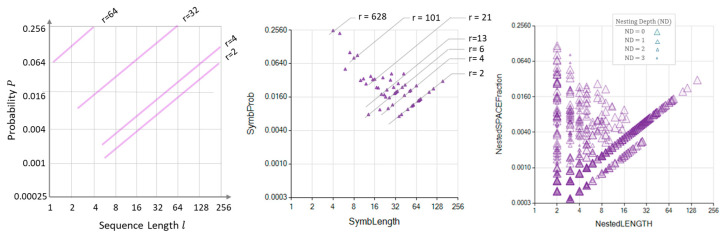
The Nested Repeated Sequence Decomposition Model (NRSDM). (**Left**) Theoretical bands reflecting the places of different sequence lengths (l) and repetitions (r) for a 10,000-char-long text. Logarithmic scales represent the sequence length and the probability, or the space fraction that the sequence length occupies in the text. (**Center**) MSIR representation of the NRSDM of a 10,000-char-long text. The aligned points reflect sequences of lengths that appear the same number of times, disregarding the nesting depth where they appear within the text. (**Right**) NPR representation of the NRSDM of a 10,000-char-long text. The nested depth of each sequence pattern expands the NPR representation of the NRSDM. Smaller bubbles represent sequences found more deeply nested into larger sequences located at a shallower depth.

Since the range of probability values is expected to be widespread, the MSIR and NPR graph axes are logarithmic. Note that the text fraction that a set sequence occupies—namely, the probability—is not independent of the sequence length. The relationship between the probability and sequence length shows graph points aligned in bands corresponding to an equal number of sequence repetitions. Figure 6 (left) illustrates the location of these equal-number-of-repetition bands for a 10000-char-long text. Figure 6 (center and right) includes examples of MSIR and NPR to show the “anatomy” helpful in interpreting these graphs.

## 3. Multiscale Integral Complexity (MSIC) and Nested Pattern Complexity (NPC)

After Shannon’s influential paper appeared in 1948 [11], complexity has been assessed as the smallest number of symbols a string needs to convey a message. In Shannon’s paper, the symbols are bits, and the name given to the smallest number of symbols is information. Counting symbols in a string is a simple task. Simple counting is not the issue. The question is what is the order—or equivalently, the patterns—that the symbols should adopt to convey the same message most efficiently? The question transforms the counting symbols problem—not a problem at all—into a rather complex optimization problem that Kolmogorov [12] later, in the context of algorithmic procedures, stated was non-solvable because there might always be a still unknown better way to organize the symbols in any message—steps in a code for the algorithmic case. Shannon did not explicitly explain how to order symbols. Nevertheless, he provided a math expression to quantify the goodness of expressing a message in binary code, which he named entropy. Shannon’s entropy formula retrieves zero entropy whenever there is a predictable order in the symbols forming the code. When there is absolute disorder, and the whole message is noise, the formula retrieves an entropy equal to one. Associating absolute order with zero entropy and total disorder with entropy = 1 is convenient; it gives us a sense of the system’s order compared with the minimum and maximum values of a system with the same number of components and two symbols in its description.

It is worth mentioning that the number of instances of each repeated sequence (*ni*) is not a distribution based on the number of text characters of the text (T). Neither is N equal to T’s length; neither is the summation of $n_{i}$ equal to N. Therefore, when computing the complexity of the process the text (T) represents, we cannot directly apply the Shannon–McMillan–Breiman Theorem [13], which offers a way to calculate entropy for ergodic processes.

In the context of this study, Shannon’s entropy measures the effectiveness of a symbol string describing a system while quantifying the noise and redundancy fractions contained. Adjusting Shannon’s expression for a symbolic diversity (d), $n_{i}$ symbols of the type i (1<=i<=d), and N symbols used in the description, we obtain Expression (8), which returns the entropy—the complexity—of a unidimensional description as the Nested Pattern Complexity (NPC):

(8) $$NPC=\sum_{i=1}^{N_{i}}\frac{n_{i}}{N}\cdot \log_{d}\frac{N}{n_{i}}.$$

Although Shannon’s entropy expression was conceived for a series of coded signals, multidimensional descriptions might be transformed into unidimensional symbol strings. Consider, for example, saving a picture in a computer’s disc. Even though the image is a two-dimensional description, the information travels to the recording media as a unidimensional list of bits. Our intuition dictates that we can convert multidimensional descriptions based on orthogonal dimensions into unidimensional descriptions. When the description is a multifaceted array of symbols, we can decompose the description space progressively until we reach a unidimensional string of symbols. However, the fractal decomposition NRSDM representing the description of the text (T) is not an orthogonal structure. Therefore, we cannot directly apply Shannon’s entropy to compute the complexity of this fractal-like description. Arguably, the number of sequences located at each nesting depth is intimately related to the structural complexity of the process that the text represents. We need to find a term to name this property. A close term is “Nestedness”, which was created by biologists to categorize structures in ecological systems. However, it refers to the distribution of species concentrations in an ecosystem [14], and its sense seems far from our objective.

In 1998, Bar-Yam [15] proposed complexity profiles to describe systems from several scaled points of view and assess complexity as a function of the scale observation level. Bar-Yam’s complexity profile depicts [16] the impact of the observation scale, retrieving the complexity associated with the system description realized at a specific observation scale. After applying **LangFractalNestedDecomp()**, the NRSDM describes the text (T) as a tree structure. The text description is modeled as significant components located immediately under the tree’s root T, containing deeper elements in a nested fashion until the most profound components—the leaves located at the deepest nested level—are reached. This tree-shaped structure is, therefore, generally fractionally dimensioned between one and two. Consequently, we cannot use Equation (8) to directly assess the tree-shaped structure’s complexity. Instead, we evaluate the complexity of this tree structure as the summation of the complexities of the nodes forming the tree. Computing the MSIC has the same numerical meaning as integrating Bar-Yam’s complexity profile function of text (T) descriptions at its nested scales. Accordingly, we refer to the complexity of this fractal-like structure as the Multiscale Scale Integral Complexity (MSIC) computed as the sum of the NPC of the tree nodes, except those which are leaves:

(9) $$MSIC=\sum_{M=1}^{Mx}{NPC}_{M}.$$

Notice the MSIC value expresses the summation of the T’s complexities, measured from all possible observation scales. Thus, texts with more than one nested level will show MSIC values exceeding one but less than the number of branch nodes in the tree.

## 4. Discretizing Numerical Series of Values

The proposed pattern detection method is a compound procedure that combines, among other aspects, setting scale limits, resolution adjustments, scale linearity changes, symbol sequence alignment, recursion, and optimization. It resembles a variational calculus applied to a discrete functional field. The approach consists of detecting repeating sequences in a long string of symbols and configuring a space of possible patterns that characterize the subject string. These prospective patterns are compared with each other to select the combination that best represents the subject string based on a defined interpretation objective.

Usually, the subject of study is a series of n real numbers. One initial step in the pattern detection process is to build an n-long sequence of elementary symbols—or chars—that represents the original numerical value series in that this symbolic string resembles, at some scale, the “rhythm of variations” of the numerical series. We refer to this step as discretization. The quality of the resemblance between the symbolic string and the series of values achieved after discretization is the subject of evaluation and depends on several parameters discussed below. Once a prospective symbolic string represents the original series of values, the method analyzes the string to extract repeated groups of chars that may constitute patterns.

### 4.1. Discretizing Scales

The method developed relies on detecting repeated sequences of elementary symbols (chars) within a long string with a certain number of symbols. When a series of real numbers represents the process studied, these values are discretized and converted to a sequence of elementary symbols. When discretizing the series of values, a type of scale may be chosen to “better absorb” the process’ nonlinearities. Appendix A.2 shows procedures for applying these discretizing scales.

### 4.2. Scale Parameter Selection

The scale parameters—type, resolution, inflection, and nonlinearity—should be selected to maximize, at least potentially, the extractable information—or negentropy—from the resulting discretized text. To measure the negentropy after applying a scale, we compute the difference between one (1), the number associated with the absence of patterns, and Shannon’s entropy [11], based on the frequency of the characters comprising the discretized text. The well-known Shannon’s entropy refers to the entropy with two different symbol values (0 and 1). To generalize Shannon’s entropy expression to a symbol diversity that might be greater than two, we follow the analysis presented by Febres and Jaffe [17], where the generalized expression must have the same symbolic diversity, or number of states, as the base of the logarithm in the Shannon’s entropy expression. In the context of this study, the diversity of chars included in the discretized text equals the scale resolution (s). Then, the base of the logarithm becomes the s, corresponding to the base of the symbolic system that we are computing. Finally, the information associated with a selected scale is as follows:

(10) $$h=1-\sum_{i=0}^{s}\frac{1}{prob\left({ScaleChar}_{i}\right)}\;\log_{s}\;prob\left({ScaleChar}_{i}\right).$$

With our goal of selecting scale parameters to maximize the negentropy in mind, we reason that a gross scale, consisting of a few chars, will be incapable of differentiating some values, producing an organized text, yet with little information to offer. The only disadvantage we see when selecting a high resolution is the potential computation burden this selection may carry. Therefore, we adopt the resolution to ensure it does not increase the computation time beyond convenience. The choice of the type of scale (linear, tangential, or hyperbolic) is carefully considered, aiming to “absorb” the process’s nonlinearity better. This nonlinearity “absorption” is reflected as an increase in the extractable information (h), defined in Expression (10). The other parameters, c, γ, and λ, are also selected to maximize the extractable information (h). 

Selecting the scale to discretize the time series values is performed before the repeated sequence recognition process. Separating the problem of choosing an appropriate scale to discretize a numerical time series provides a relevant advantage because it does not augment the method’s algorithmic complexity.

## 5. Examples of Pattern Descriptions of Processes (Series of Values)

In this section, numeric series are discretized to form texts representing the original series of values. After this series representation change, we obtain the NRSDM that describes some of the characteristic properties of the examples below. 

### 5.1. The May’s Logistic Map

In 1976, the biologist Robert May published a paper [18] studying population growth dynamics. He paid particular attention to the model $x_{n+1}=\rho \;x_{n}\left(1-x_{n}\right)$, where $x_{n+1}$ is the population at stage n+1 recursively computed in terms of the previous population ($x_{n}$) and a growth parameter (ρ). As this model is recognized, May’s logistic map has become a reference to illustrate the transition of systems dynamics from stable to chaotic. In this section, we apply our method to identify the periods at the regimes of the model experiments at different values of ρ.

**Figure 7:**
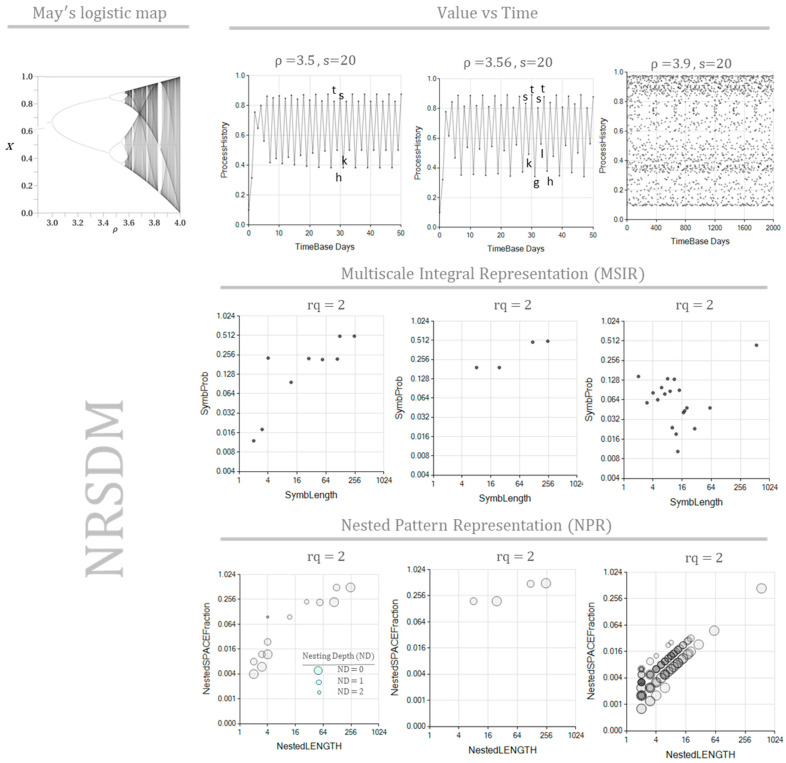
The MSIR and NPR of May’s logistic equation. Patterns detected for May’s logistic map are shown in the upper-left corner. The **top** graphs show the development of equation $x_{n+1}=\rho \;x_{n}\;(1-x_{n})$ for different parameter values: ρ=3.5, ρ=3.56, and ρ=3.9. The **bottom** graphs show the corresponding probability–length decomposition, determined using the scale resolution s=20 and r=2 repetitions.

We tested May’s equation regimes at the parameters ρ=3.5, ρ=3.56, and ρ=3.9 to verify that the method’s application returns proper results. A tangential scale of resolution (s=20) represents each process value with letters from “a” to “t” (the 20th letter), covering the smallest values to the largest ones. Table 1 shows the conditions for these May’s equation tests. Figure 7 shows how May’s equation dramatically changes behavior when these parameter values change, so we expect to detect several distinctive patterns. The three simulations begin with a transient time that does not repeat any cycle. However, after a long enough time, the process settles in repetitive sequences of values. For ρ=3.5, the simulation repeats cycles around the four-value sequence “t*shk*”, which is the basis of larger (up to 249 values) repetitive sequences detected. For ρ=3.56, the eight-value sequence “s*ktgslth*” is the basis of larger repetitive sequences detected, up to 248 values long. Sequences as short as two values long appear as repetitive sequences in the transient zone.

As May stated [18], model $x_{n+1}=\rho \;x_{n}\left(1-x_{n}\right)$ enters a chaotic region for values ρ≥3.9. Nevertheless, the process continues to show stable cycles. Consequently, after the initial transient stage, we should expect repetitive sequences to fill the search space. This time, however, the length of the primary repeated sequence is 548, which is much longer than in previous cases. Note we had to extend the simulation to 2501 time steps to provide conditions to detect this large repetitive sequence. At this point, we highlight the algorithm’s capacity to recognize such a long, repetitive sequence. In [18], May mentioned that the process of $x_{n+1}=\rho \;x_{n}\left(1-x_{n}\right)$, although chaotic, shall have a long, stable cycle; the process is considered chaotic because of the infinite number of different cycles it can exhibit.

### 5.2. Lorentz’s Equations

Lorentz’s equations are a typical example of a bounded three-dimensional chaotic system. The status of a system governed by Lorentz’s equations orbits around two attractors but never exactly repeats a previously followed trajectory. Nevertheless, if differences between the values of two states are small enough to be considered irrelevant, several “practically equal” long sequences of states will appear. Lorentz’s equations are as follows:

(11) $$\frac{dx}{dt}=\sigma \;\left(y\left(t\right)-x(t)\right),$$

$$\frac{dy}{dt}=\rho \;x\left(t\right)-y\left(t\right)-x\left(t\right)\;z\left(t\right),$$

$$\frac{dz}{dt}=x\left(t\right)\;y\left(t\right)-\beta \;z\left(t\right).$$

**Figure 8:**
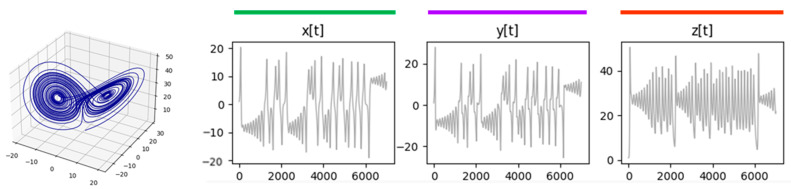
Simulation of Lorentz equations represented in a three-dimensional space, and the process trajectory for each dimensional axis.

The set of Lorentz equations (11) includes the state positions (x(t), y(t), z(t)) and their corresponding derivatives ($\frac{dx}{dt},\frac{dy}{dt},$ and $\frac{dz}{dt}$). The Lorentz equation model, therefore, is a six-degrees-of-freedom system living in a three-dimensional space. We extracted repeated trajectory segments considering Lorentz process positions. Thus, we obtained the NRSDM of the discretized series x(t), y(t), $z\left(t\right)$, leaving the analysis of the derivatives for a future study. Figure 8 shows the simulation’s results obtained after discretizing time (t) in Equation (11) for times from t=0 to t=35, and a Δt=0.005. The initial conditions were as follows: x(0)=1, y(0)=1, and $z\left(0\right)=1$.

**Figure 9:**
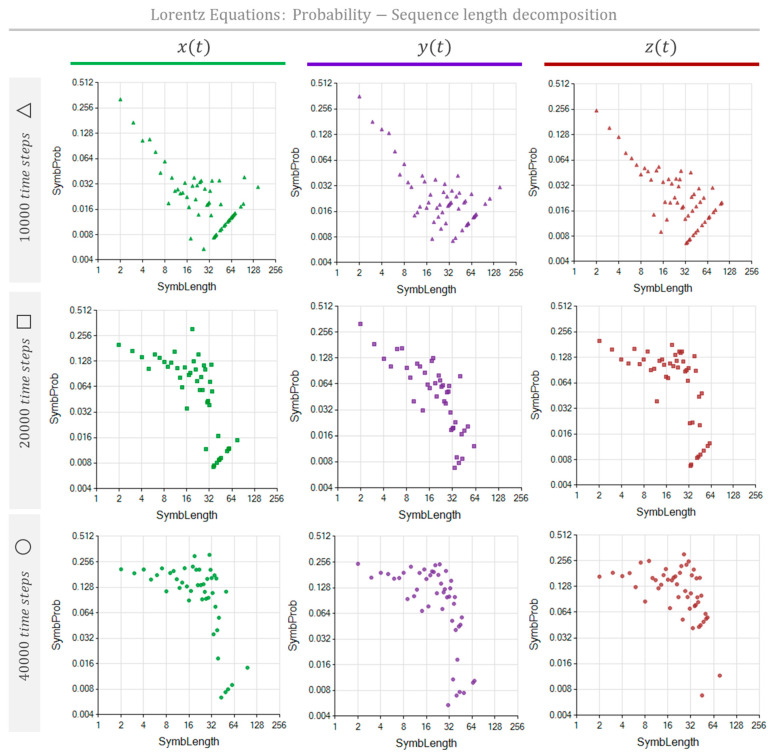
The MSIR of the Lorentz equation process, simulated with 10,000 time steps (**top**), 20,000 time steps (**middle**), and 40,000 time steps (**bottom**), is characterized by the probability of repeating sequences as a function of the sequence length. The graphs show the sequence length probability distributions of the process described as x(t), y(t), and $z\left(t\right)$. Probability distributions are green, violet, and red for x(t), y(t), and $z\left(t\right)$, respectively.

**Figure 10:**
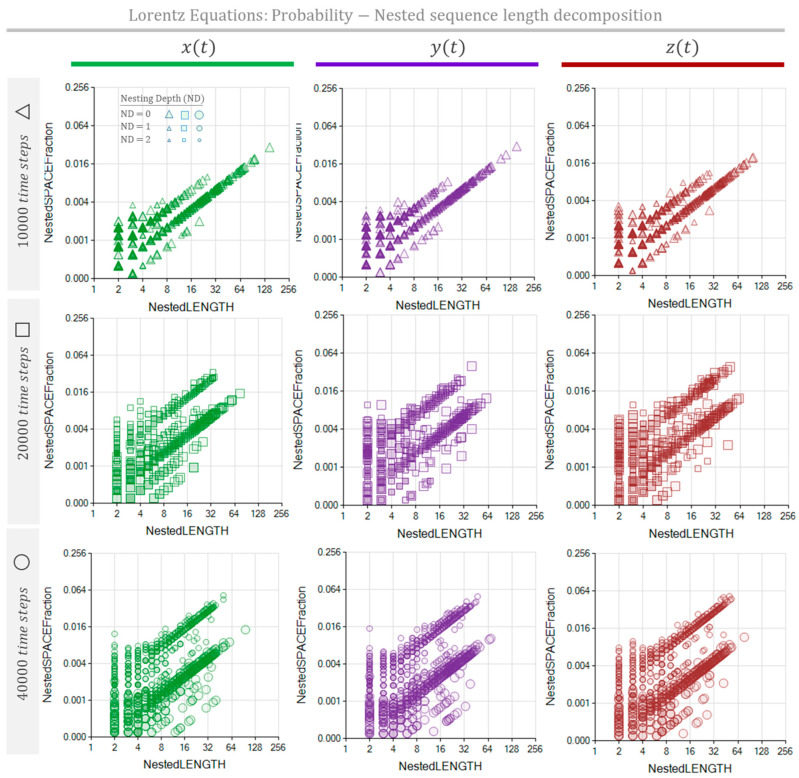
The NPR of the Lorentz equation process, simulated with 10,000 time steps (**top**), 20,000 time steps (**middle**), and 40,000 time steps (**bottom**), is characterized by the probability of repeating sequences as a function of the sequence length. The graphs show the sequence length probability distributions of the process described as x(t), y(t), and $z\left(t\right)$. Probability distributions are green, violet, and red for x(t), y(t), and $z\left(t\right)$, respectively.

Table 2 shows the parameters we used to simulate the series x(t), y(t), and $z\left(t\right)$. The Lorentz system was modeled for three different time lengths, accomplished by applying the method with 10,000, 20,000, and 40,000 time steps. Lorentz equations have periodic trajectories orbiting around fixed attractors. However, these cycles are unstable [19,20], meaning that there will not be any cycle that ends precisely in the position and velocity state at which the cycle began. Consequently, contrary to May’s formerly presented equation, we do not expect to identify repetitive sequences that fill an entire orbit loop. Figure 9 shows the MSIR of the NRSDM as the fraction of space occupied by equally long repeated sequences, independently of their nesting depth. Figure 10 shows the NPR of the NRSDM unfolding of these space fractions by representing the equally long sequences with different bubbles at their corresponding nesting depths.

Figure 9 shows similar graphs corresponding to the same data length, revealing that these three processes are interlaced. However, when the data length increases, it produces a noticeable change in the sequence repetition nested patterns, reflecting the impact of the scope size on the text interpretation process. Comparing the shape of the dot clouds, we see a transformation as the simulated time increases. The cloud starts from an approximated triangular distribution at 10,000 time-step simulations and progressively becomes a bow-shaped cloud for 40,000 time-step simulations. As the simulation time enlarges, the maximum probability sequence length goes from two (at 10,000 time-step simulations) up to nearly 30 (at 40,000 time-step simulations). While x(t), y(t), and $z\left(t\right)$ share similar graph pattern evolutions, suggesting this is not an accident, we are still determining the origin of this cloud-shape transformation. A hypothesis is that the cloud-shape transformation reveals the impact of the transient stages from arbitrary initial conditions. Should this be the case, the MSIR of the NRSDM would be a tool to assess how far from its natural—non-transient—behavior a complex process is.

The NPR in Figure 10 shows the expected increase in bubbles as the simulation length grows. We observe an additional effect: a second lobe emerges in the bubble cloud as the simulation length increases. According to the bubble sizes, the higher-probability lobe is a compound of sequences with a more deeply nested location. The emergence of multiple lobes reveals the process’s internal structure characteristics when the transient stage is over and stable conditions dominate the process.

### 5.3. Non-Periodic Trajectory Processes: Stock Market Time Series

The trajectory of some processes can drift arbitrarily far from any fixed reference. In these cases, the time series do not exhibit limits in their values, and detecting periodic behaviors is hard at any time scale, accentuating the chaotic appearance of the process and adding difficulty to the information extraction from these descriptions. Economic indexes generally are within these categories of limitless trajectories. Besides inflation, which has an arguably zero reference value, financial time series usually rely on prices and indexes whose values seem to not orbit any attractor, at least not for a long epoch. Economic indexes, however, do have momentum.

Studying the derivative of the values, rather than the original series values, can be a game-changer. This change adds an attractor to the studied trajectory, producing a bounded index trajectory with likely recurring harmonic behaviors. This approach significantly enhances our possibilities for information extraction.

**Figure 11:**
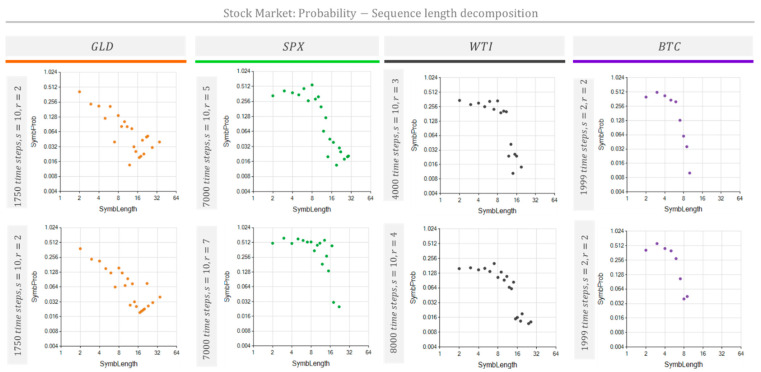
The MSIR of the NRSDM for several stock market indexes. Probability vs. sequence length decomposition diagrams for four representative market time series: Gold (GLD), the Standard and Poor Index (SPX), West Texas Intermediate (WTI), and BitCoin (BTC). Each diagram corresponds to the daily value change in the stock and was built with the scale and modeling conditions indicated in the left vertical bar next to each graph and Table 3.

Figure 10 and Figure 11 show the NPR and the MSIR of the NRSDM for four typical stock market indexes and prices: the Gold (GLD) price, Standard and Poor 500 Index (SPX), West Texas Intermediate (WTI) oil price, and Bitcoin (BTC) price. Table 3 shows the data and the parameters used to obtain the results in Figure 10 and Figure 11. The objective is to evaluate non-periodic processes and to extract information from these usually-considered-chaotic time series. The results shown in Figure 10 and Figure 11 correspond to the daily variation in the series shown in Appendix A.2.

Figure 10 and Figure 11 show that repeated sequences occupy the space fraction according to length. As expected, the number of repetitions dramatically grows as the sequence length shortens. This effect drives the space fraction for short sequences up to several orders of magnitude above the space fraction corresponding to longer repeated sequences.

**Figure 12:**
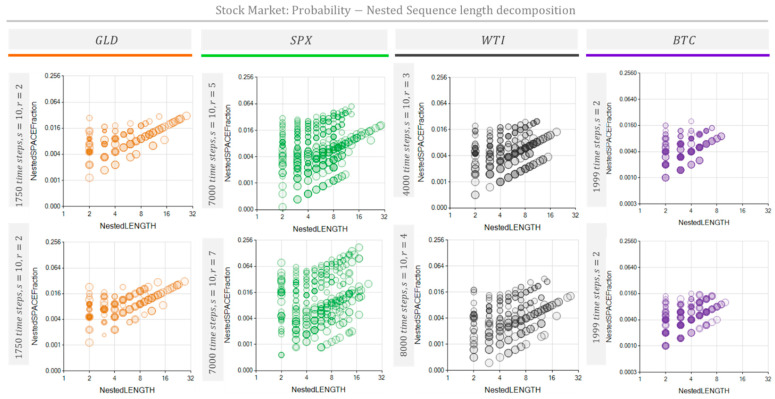
The NPR of several stock market indexes. Probability vs. nested sequence length decomposition diagrams for four representative market time series: Gold (GLD), the Standard and Poor Index (SPX), West Texas Intermediate (WTI), and BitCoin (BTC). Each decomposition diagram was built with the conditions indicated in the left vertical bar next to each graph.

The repetition patterns detected for these market prices reveal characteristic differences among the stock market processes assessed. These processes’ fingerprints are visible in the space fraction occupied by repetitive sequences included in Figure 11 and the unfolded nested repeated sequences shown in Figure 12. Additionally, Figure 10 and Figure 12 show the BTC price change tendency to repeat shorter cycles of about ten days (represented by ten chars) than its counterparts GLD, the SPX, and WTI, which exhibit cycles of nearly 30 days.

Analyzing the shapes of the bubble clouds in Figure 10 and Figure 11, we observed characteristics similar to those discussed in Figure 8 and Figure 9. The MSIRs (Figure 11) of the SPX, WTI, and BTC exhibit a bow-shaped distribution, suggesting an established process. On the contrary, the MSIR for GLD shows a triangular dot cloud, which we hypothesized is due to a process going through a transient condition. Meanwhile, the NPRs (Figure 12) of the SPX, WTI, and BTC show two separated bubble clouds presenting different nesting levels. In contrast, the NPR for GLD does not show multiple bubble clouds, suggesting that a transient stage dominates the process.

## 6. Results, Summary, and Discussion

We present a method based on an algorithm capable of detecting patterns in long texts. By recursively applying this algorithm, the procedure achieves the text decomposition in nested symbolic sequences, resulting in the NRSDM. The procedure functions on the base of the dimensionality reduction. Thus, we can think about the method as transforming a text object, whose dimensionality is of the order of the word diversity, into a lower-dimensional structure. The transformation follows a parameter set to define a specific process interpretation. Among the most relevant of these parameters are the following:

- The discretization scale (s). Resolution: alphabet size. Scale’s nonlinearity: tangential, hyperbolic, linear;
- Repetitions ($r_{q}$): the number of instances a sequence must appear to be considered a repeated sequence within the context of our method;
- The text length (L): text segment selected from the original full text.

Processing indefinitely long texts with these algorithms is prohibitive; the overall complexity of the algorithms is $O({T.Length}^{3})$. In our experiments, we used an Intel i7 processor. The 40,000-character texts of the Lorentz simulations were the longest texts we had time to process. To shorten the execution time for 40,000-character texts, we had to increase the repetitions required ($r_{q}$).

The structure resulting from this transformation becomes the NRSDM we use to study the process by interpreting and representing it. In this document, we characterized three different types of time series: May’s equation, Lorentz equations, and several stock market price evolutions in time. We obtained two representations for each process: the MSIR and the NPR. The method and its resulting representations offer possibilities for further comparing processes’ characteristics, extracting synthetic descriptions, and a better understanding of systems’ behavior, thereby providing ways to enhance data for modeling and projection purposes.

The MSIR’s dimensionality equals the number of different sequence lengths found. These sequence lengths are the domain of the graphs shown in Figure 6 (middle row), Figure 9 and Figure 11. Thus, each dot in these graphs may represent several sequences sharing their lengths. Hence, the number of dots is considerably less than the number of sequences. The dimensionality of the counterpart representation, the NPR, equals the number of different sequence lengths and the number of nesting depths found in the NRSDM decomposition. The domain of the graphs shown in Figure 6 (bottom row), Figure 10 and Figure 12 represent the sequence lengths, while the size of the bubbles represents the nesting level of each sequence. Consequently, NPR graphs exhibit a denser cloud of bubbles than the MSIR.

The MSIR and the NPR are the pinnacle results of this study. These representations constitute visual fingerprints of the complex processes coded as unidimensional texts. Comparing the aspects of these graphs is already a possibility for contrasting processes. The structural distances among processes emerge in these graphs as noticeable differences. Focusing on the MSIR of Lorentz’s equations, we observe a progressive dot-cloud shape change from triangular for 10,000 time-step simulations to an accentuated bow dot-cloud shape for 40,000 time-step simulations. Since this progressive behavior occurred in dimensions x, y, and z of Lorentz’s process, we hypothesized that the “natural, or typical” shape of the MSIR of the Lorentz process is a bow-shaped cloud of dots, associating the triangular shape with the transient effects when the process is still near non-natural initial conditions and far from its fully developed stable behavior. Changing our attention to the NPR graphs in Figure 9 and Figure 11, we notice significant differences between the NPR of Lorentz’s equations for 10,000 time-step simulations versus those for the 20,000 and 40,000 time-step simulations. These last simulations exhibit multiple bubble clouds consistent with the graphic emergence of nested repeated sequence structures.

**Figure A2:**
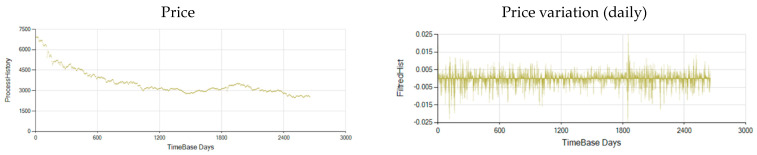
Market GLD price and daily variations registered from 6,756.10 USD/Onz on 10/19/2012 (day 0) to USD 2,563.99 on 6/1/2023 (day 2656).

Inspecting the MSIR graphs for stock market indexes in Figure 11, we note that GLD shows a triangular dot-cloud shape, while SPX, the WTI, and BTC show a bow dot-cloud shape. The NPR graphs for stock market indexes in Figure 12 also show a single bubble cloud for GLD. Interestingly, from the four stock market indexes addressed, GLD shows the most dramatic and noticeable change in the records we used here. See the market GLD price and daily variations registered in Figure A2 in Appendix A.2; note the oscillation pattern change near the TimeBase Day 1900.

We did not perform a statistical analysis of the connections between the shapes of the MSIR and NPR graphs, as this would have gone beyond the scope of this study. Therefore, significant speculation exists about the system properties and conditions derived from these graphs’ shapes. However, these early results suggest we are just beginning to develop an analysis method with promising potential for studying complex systems.

Left to the end is the direct result obtained from the method: assessing the complexities of the processes studied. Clearly, the results show the complexity ranking of these systems. GLD and BTC, for example, carry less information than the SPX and WTI do. These sorts of comparisons are a result of the method. Perhaps more important is the possibility of obtaining these complexity assessments for the two modes of systems described here: i) the synthetic math model for the dynamic of May and Lorentz processes, and ii) the extended system response to the surrounding, mostly unknown conditions represented by the stock market processes.

## 7. Conclusions

This study introduces an algorithmic method for decomposing a unidimensional series of values into long repeated sequences. The procedure simplifies the search space while keeping the text’s structural essence, making the nested pattern detection computationally feasible. 

The NRSDM is a valuable tool for characterizing long texts that may represent the behavior of unidimensional processes. The possibility of representing the tendency of specific sequence lengths to exist at certain nested depths may become, in further studies, an even more powerful tool to study and compare the system structure and behavior. These procedures are promising tools for characterizing and representing complex systems. By detecting the hidden nested structures within any complex system and performing MSIR and NPR, we identify ways to quantify the nested complexity of systems and the degree of stability conditions the process is going through.

## Figures and Tables

**Table 1:** Pattern detection in May’s logistic map equation: parameters and results.

Process	Scale: Type: Res/Inflect/Param	Sample Length	$r_{q}$: Repetitions Required	Repetitions Shrtest Symb.	Shortest/Longest Repeated Seq.	Complexity: MSIC/NPC	Shown in Figure 7 Row Col/Row Col
*ρ* = 3.5	T: 20/10/0.01	1001	2	2	249	2.02/0.844	Cntr Left/Bttm Left
*ρ* = 3.56	T: 20/10/0.01	1001	2	2	248	1.04/0.729	Cntr Cntr/Bttm Cntr
*ρ* = 3.9	T: 20/10/0.01	2501	2	2	548	8.32/0.980	Cntr Rght/Bttm Right

**Table 2:** Pattern detection in Lorentz equation system: parameters and results.

Process	Scale: Type: Res/Inflect/Param	Sample Length	$r_{q}$: Repetitions Required	Repetitions\Symb.	Longest Sequence	Complexity: MSIC/NPC	Shown in Figure 9 and Figure 10
Lorentz’s x(t)	T: 22/11/0.01	10,000	2	2	147	77.527/0.984	Green Top
	T: 22/11/0.01	20,000	4	2	75	6.202/0.957	Green Middle
	T: 22/11/0.01	40,000	6	2	96	2.113/0.939	Green Bottom
Lorentz’s y(t)	T: 22/11/0.01	10,000	2	2	154	89.734/0.982	Violet Top
	T: 22/11/0.01	20,000	4	2	61	11.271/0.954	Violet Middle
	T: 22/11/0.01	40,000	6	2	69	2.998/0.940	Violet Bottom
Lorentz’s z(t)	T: 22/11/0.01	10,000	2	2	100	61.578/0.985	Red Top
	T: 22/11/0.01	20,000	4	2	62	4.115/0.957	Red Middle
	T: 22/11/0.01	40,000	6	2	77	1.349/0.941	Red Bottom

**Table 3:** Pattern detection in stock market time series: parameters and results.

Process	Scale: Type: Res/Inflect/Param	Sample Length	$r_{q}$: Repetitions Required	Repetitions Shrtest Symb.	Longest Sequence	Complexity: MSIC/NPC	Shown in Figure 11 and Figure 12
Daily change in GLD price	T: 10/5/0.01	1750	2	2	35	23.549/0.967	Orange
	T: 10/5/0.01	1750	2	2	35	22.706/0.968	
Daily change in SPX index	T: 10/5/0.01	7000	5	2	29	2.988/0.926	Green
	T: 10/5/0.01	7000	7	2	22	1.200/0.905	
Daily change in WTI price	T: 10/5/0.01	4000	3	2	19	2.824/0.934	Black
	T: 10/5/0.01	8000	4	2	26	1.717/0.950	
Daily change in BTC value	T: 16/8/0.01	1999	2	2	9	11.582/0.974	Violet
	T: 16/8/0.01	1999	2	2	10	12.141/0.976	

**Figure A1:**
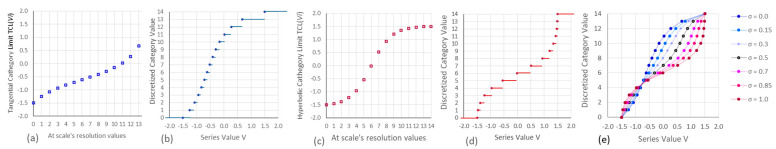
Properly adjusted discrete scales can absorb the modeled series’ nonlinearity. Graphs (**a**,**b**) show a tangential discrete scale with resolution at s=15, extreme values at $V_{mn}$ = −1.5 and $V_{Mx}$ = 1.5, the scale inflection point at c=6, and parameter γ=0.82. Graph (**a**) shows the Tangential Category Limits computed as a function of each possible value of the scale’s resolution applying Equation (A1). Graph (**b**) shows the category discretely assigned to real series values in the horizontal axis. Graphs (**c**,**d**) show a hyperbolic discrete scale with resolution at s=15, extreme values at $V_{mn}$ = −1.5 and $V_{Mx}$ = 1.5, the scale inflection point at c=6, and parameter λ=1.7. Graph (**c**) shows the Tangential Category Limits computed as a function of each possible value of the scale’s resolution applying Equation (A2). Graph (**d**) shows the category discretely assigned to real series values in the horizontal axis. Graph (**e**) shows the spectrum of discretizing scales obtained by linearly combining the two former scales according to a weight (σ).

**Figure A3:**
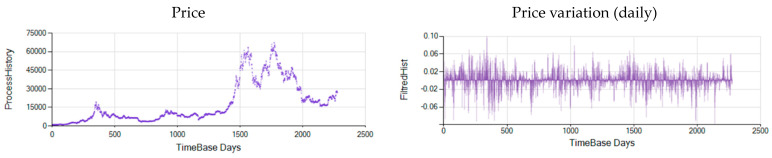
Market BTC price and daily variations registered from USD 999.40 on 1/1/2017 (day 0) to USD 42,315.00 on 12/26/2023 (day 2553).

**Figure A4:**
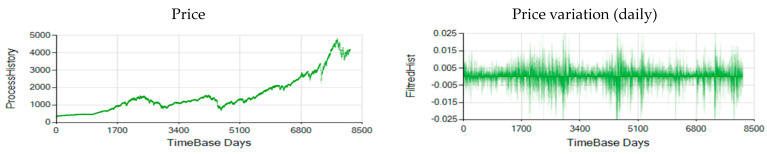
Market SBX index and daily variations registered from a value of 326.43 on 1/2/1991 (day 0) to 4,221.02 on 6/1/2023 (day 8165).

**Figure A5:**
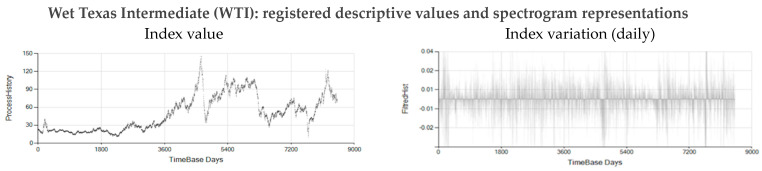
Market WTI price and daily variations registered from 22.89 USD/Brrl on 1/2/1990 (day 0) to 73.38 USD/Brrl on 6/4/2023 (day 8516).

**Figure A6:**
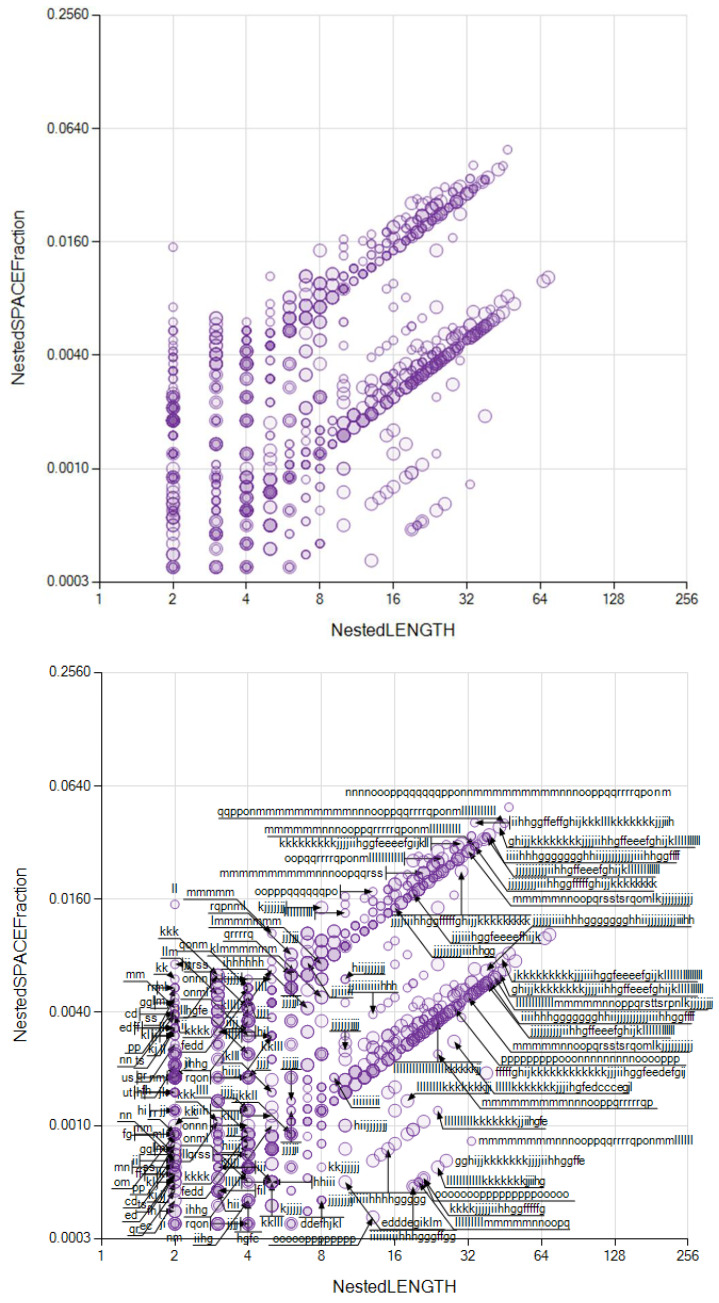
The NPR of the NRSDM of the dimension y(t) of the Lorentz equations simulated with 40,000 time steps. Probability of repeating sequences vs. repeated sequence length. The graphs show the sequence length probability distributions of the process described as y(t). The tags indicating each repeated sequence are hidden in the top figure for clarity.

**Figure A7:**
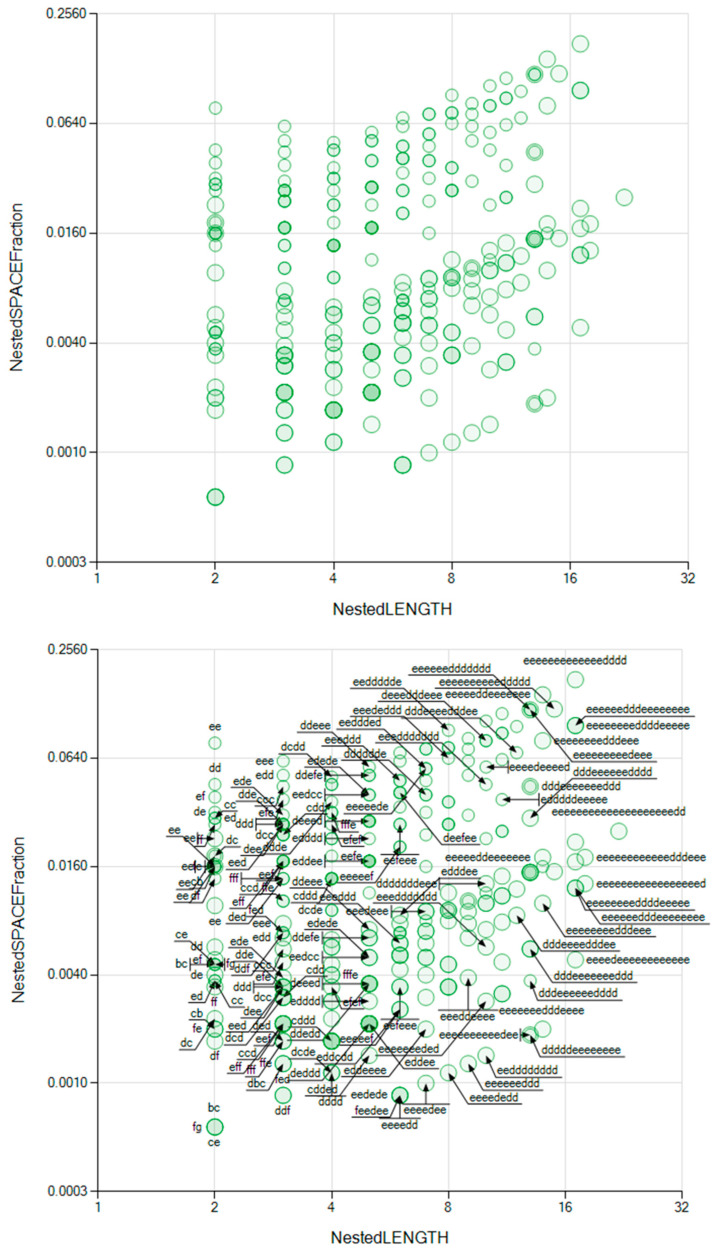
The NPR of the NRSDM of the SPX. Probability of repeating sequences vs. repeated sequence length. The graphs show the sequence length probability distributions of the process described as y(t). The tags indicating each repeated sequence are hidden in the top figure for clarity.

## Data Availability

The data descripting May’s and Lorentz’s processes was obtain from simulations performed as part of this study. The data describing the history of prices of gold, West Texas Intermediate oil, Standard and Poor index, and the Bit Coin is public. The values used were obtained from https://www.investing.com.

## Appendix A

### Appendix A.1. Discretizing Procedures

Consider a series (V) formed by the actual values ($V_{i}$), where the minimum and maximum values are $V_{mn}$ and $V_{mx}$, respectively. The objective is to assign a category or a symbolic discrete representation of each value ($V_{i}$) of the series. The categories belong to a scale of resolution (s) that is ordered according to the corresponding values ($V_{i}$). Several scales work as transformers to fit the nonlinearity of series (***V***) values better. Some types of scales are presented below.

#### Appendix A.1.1. The Linear Discrete Scale

The linear scale is regarded as the most intuitive scale for interpreting and reasoning about process dynamics. It consists of splitting the range of process values into equally spaced category values. The number of categories is called s, the scale’s resolution.

#### Appendix A.1.2. The Tangential Discrete Scale

Function TCL(), shown in Expression (A.11), returns the upper-limit value ($TCL\left(V_{i}\right)$) of the discretized category corresponding to the value ($V_{i}$). The discrete scale’s (TCL()) shape resembles the nonlinearity of the tangent function. Thus, large negative values appear when the argument approaches −π2, and significant positive values appear when the argument approaches π2. Parameter c allows the control of the scale value where the tangent infection point is located. The tangential discrete scale has a resolution (s), meaning that all the values ($V_{i}$) in the series are assigned to one of the s segments (or categories) within the scale:

(A1) $$TCL\left(V_{i}\right)=V_{mn}+D\frac{\tan\left(E_{\left(i\right)}\right)-\tan\left(E_{\left(mn\right)}\right)}{\tan\left(E_{\left(Mx\right)}\right)-\tan\left(E_{\left(mn\right)}\right)}\;$$

$$E_{\left(i\right)}=\left\{\begin{array}{c} \;\frac{{\;\gamma \;G}_{\left(i\right)}\;\pi \;}{G_{\left(Mx\right)}\;2}\;,\;if\;\left|V_{Mx}\right|\geq \left|V_{mn}\right|\;,\\ x\\ -\frac{{\;\gamma \;G}_{\left(i\right)}\;\pi \;}{G_{\left(mn\right)}\;2}\;,\;otherwise\;, \end{array}\right.$$

$$G_{\left(i\right)}=V_{\left(i\right)}+\left(\frac{s-1}{2}-c\right)\left(\frac{D}{r-1}\right)=V_{\left(i\right)}+D\left(\frac{1}{2}-\frac{c}{s-1}\right)$$

$$V_{\left(i\right)}=V_{mn}+\frac{s-1}{D}\;i\;,\;i=0\;,\;1\;,\;\ldots ,\;s-1,$$

$$D=V_{Mx}-V_{mn}.$$

Figure A1a,b show the results of applying Equation (A1) to a tangential discrete scale with resolution of s=15 and a series of values categorized with values between $V_{mn}=-1.5$ and $V_{Mx}=1.5$, parameter γ=0.82, and the location of the inflection point at the scale category c=5.

#### Appendix A.1.3. The Hyperbolic Tangent Discrete Scale

Function HCL() shown in Expression (A.12) returns the upper-limit value ($HCL\left(V_{i}\right)$) of the discretized category corresponding to the value ($V_{i}$) The discrete scale’s (HCL()) shape resembles the hyperbolic-tangent function’s nonlinearity. Thus, asymptotically, the maximum scale value ($V_{Mx}$) is reached when the maximum value of the argument is s=n−1, and asymptotically, the minimum scale value ($V_{mn}$) is reached when the argument is s=0. The hyperbolic-tangent discrete scale has a resolution (s), meaning that all the values ($V_{i}$) in the series are assigned to one of the scale’s s segments (or categories); the parameter λ and the location of the inflection point (c) control the shape of the function HCL():

(A2) $$HCL(V_{i})=V_{mn}+\frac{D\;(F_{\left(i\right)}-F_{mn})}{\left(F_{Mx}-F_{mn}\right)}\;$$

$$F_{\left(i\right)}=\frac{D}{2}\;\tanh\left(\lambda \;G_{\left(i\right)}\right)$$

$$G_{\left(i\right)}=V_{i}+\left(\frac{r-1}{2}-c\right)\left(\frac{D}{s-1}\right)=V_{i}+D\left(\frac{1}{2}-\frac{c}{s-1}\right)$$

$$V_{\left(i\right)}=V_{mn}+\frac{s-1}{D}\;i\;,\;i=0\;,\;1\;,\;\ldots ,\;s-1,$$

$$D=V_{Mx}-V_{mn}.$$

Figure A1c,d show the results of applying Equation (A2) to a hyperbolic-tangent discrete scale with a resolution of s=15 and a series of values to be categorized with values located between $V_{mn}=-1.5$ and $V_{Mx}=1.5$, parameter λ=1.7, and the location of the inflection point at the scale category c=6.

#### Appendix A.1.4. The Combined Discrete Scale

The tangential and hyperbolic scales can be linearly combined to produce intermediate scales to reach a good fit between the modeled process and the scale representing it. Figure A1e shows different scales obtained by mixing the scales in Figure A1b,d with different weights. Combined discrete scales may be useful when automating the scale selection to discretize the process.
